# Longitudinal analysis of human plasma biomarkers for Alzheimer's disease: Phosphorylated Tau‐217, Phosphorylated Tau‐181, and Glial fibrillation acidic protein

**DOI:** 10.1002/alz70856_102320

**Published:** 2025-12-25

**Authors:** MayaRae N Mugosa, Jefferson W Kinney, Lorenzo Gabriel Pasia, Mason Jonah, Isabella Hou, Aaron Ritter

**Affiliations:** ^1^ UNLV, Las Vegas, NV, USA; ^2^ University of Nevada Las Vegas, Las Vegas, NV, USA; ^3^ UNR, Reno, NV, USA; ^4^ Cleveland Clinic, Las Vegas, NV, USA; ^5^ Hoag Medical Center, Newport Beach, CA, USA

## Abstract

**Background:**

Alzheimer's disease (AD) is a progressive neurodegenerative disease affecting memory and cognitive processes, making it increasingly difficult for individuals to perform daily tasks as the disease progresses. Early diagnosis and tracking of disease progression is crucial for optimal treatment and support. Current diagnostic methods for AD include positron emission tomography (PET), computed tomography (CT), magnetic resonance imaging (MRI), evaluation of cerebrospinal fluid (CSF) and other invasive and expensive tools. Blood biomarkers provide more cost‐effective and scalable assessment and disease progression.

**Methods:**

We have previously evaluated human plasma concentrations of glial fibrillary acidic protein (GFAP) and phosphorylated tau‐181 (ptTau181) using the single molecular array (SIMOA) Quanterix instrument. Using chemiluminescent enzyme immunoassay (CLEIA) technology on the Lumipulse G1200 platform, we evaluated concentrations in human plasma for phosphorylated tau‐217 (pTau217). Protocols for each corresponding protein kit were followed without modification.

**Results:**

Concentrations of these proteins were analyzed for differences between disease states over a two year difference to identify potential significant disease progression. Despite having no statistical significance, the longitudinal analysis suggests a consistent trend over time, with a gradual increase in target concentrations of some candidate biomarkers.

**Conclusions:**

The longitudinal analysis revealed intriguing trends that warrant further investigation with a larger sample size. Continued pursuit of pTau217 would be beneficial, given its strong correlation with amyloid status, which suggests potential as a valuable biomarker for AD disease progression.

